# Increased Artemis levels confer radioresistance to both high and low LET radiation exposures

**DOI:** 10.1186/1748-717X-7-96

**Published:** 2012-06-19

**Authors:** Deepa M Sridharan, Mary K Whalen, Donna Almendrala, Francis A Cucinotta, Misako Kawahara, Steven M Yannone, Janice M Pluth

**Affiliations:** 1 Life Sciences Division, Lawrence Berkeley National Laboratory, Berkeley, CA 94720, USA; 2 NASA, Lyndon B. Johnson Space Center, 2101 NASA Parkway, Houston, TX 77058, USA

**Keywords:** Artemis, Radioresistance, High LET radiation

## Abstract

**Background:**

Artemis has a defined role in V(D)J recombination and has been implicated in the repair of radiation induced double-strand breaks. However the exact function(s) of Artemis in DNA repair and its preferred substrate(s) *in vivo* remain undefined. Our previous work suggests that Artemis is important for the repair of complex DNA damage like that inflicted by high Linear Energy Transfer (LET) radiation. To establish the contribution of Artemis in repairing DNA damage caused by various radiation qualities, we evaluated the effect of over-expressing Artemis on cell survival, DNA repair, and cell cycle arrest after exposure to high and low LET radiation.

**Results:**

Our data reveal that Artemis over-expression confers marked radioprotection against both types of radiation, although the radioprotective effect was greater following high LET radiation. Inhibitor studies reveal that the radioprotection imparted by Artemis is primarily dependent on DNA-PK activity, and to a lesser extent on ATM kinase activity. Together, these data suggest a DNA-PK dependent role for Artemis in the repair of complex DNA damage.

**Conclusions:**

These findings indicate that Artemis levels significantly influence radiation toxicity in human cells and suggest that Artemis inhibition could be a practical target for adjuvant cancer therapies.

## Background

Radiation therapy is one of the most broadly prescribed treatments for malignancy. However, the reasons for the wide inter-individual variability in response to radiation therapy are not apparent. Identifying factors that predict resistance to therapy is an area of intense research that holds promise to facilitate personalized treatments and improve outcomes. Here we have identified elevated Artemis levels as being a powerful determinant of radiation resistance in human cells.

Defects in the Artemis gene were found to be causal in radiosensitive severe combined immunodeficiency disorders in a subset of Japanese, European, and Native American SCID patients [1,2]. Individuals lacking functional Artemis are extremely sensitive to radiation and cannot generate mature B or T cells due to incomplete V(D)J recombination which results in immunodeficiency [1]. Artemis has been established as an essential hairpin-cleavage enzyme in V(D)J recombination, however its exact role in radioresistance has been more difficult to define. Artemis is thought to function in non-homologous end joining (NHEJ), the primary repair pathway for double-strand breaks (DSBs) in G1 phase of the cell cycle. Artemis has been implicated as playing a role in this pathway because of the radiosensitivity associated with Artemis defects and the functional and physical interactions between Artemis and the DNA-dependent protein kinase (DNA-PK). DNA-PK plays a central role in NHEJ repair, phosphorylating itself and other proteins during the repair process and acting as a scaffold for repair protein assembly on DNA ends [3]. In the presence of DNA-PK, Artemis acquires a DNA endonuclease activity which is important for its role in hairpin processing in V(D)J recombination and perhaps key in processing specific radiation-induced substrates in NHEJ [4-7]. Studies have shown that DNA-PKcs recruits Artemis to DSBs within the chromatin [8,9], aligns the ends of the DSB to enable Artemis-mediated processing [4], and that together they can cleave blocked DNA termini [10,11].

A model has been proposed based on accumulated data that Artemis phosphorylation is not required for its endonuclease repair function, but rather DNA-PKcs autophosphorylation is, and that the latter modification allows conformational changes in the DNA that are necessary for Artemis’ intra-stand incision capability [4]. ATM, DNA-PKcs and ATR (ATM related) have been shown to phosphorylate Artemis *in vitro*, evidence suggests ATM is the primary *in vivo* kinase phosphorylating Artemis following ionizing radiation [12-17], however other studies reveal Artemis *activity* is entirely dependent on its interaction with DNA-PK [9]. Phosphorylation of Artemis by DNA-PKcs or ATM is not required for the endonuclease activity of Artemis, and has been shown to be uncoupled from its repair function [4], although it does influence cell cycle progression [12]. Studies suggest that phosphorylation of Artemis by ATM is a negative regulator of G2/M checkpoint, and that upon repair Artemis is de-phosphorylated [8,12,16]. Despite the role of Artemis phosphorylation on cell cycle progression, there does not appear to be a clear indication that phosphorylation is key to its repair function.

An increased sensitivity to agents that induce more complex damage as compared to simple DSB inducing agents suggested an important role for Artemis in the repair of complex double strand breaks [10,13]. Previously we had shown Artemis defective cells exhibit elevated radiosensitivity, long-term unresolved damage demonstrated by persistent G1 and G2/M arrest, and a subtle DSB repair defect, exhibiting ~2 fold more γH2AX foci as compared to controls [16]. These results are consistent with earlier findings showing that γH2AX levels can remain elevated up to 14 days following alpha particle radiation exposure in Artemis defective-cells [13]. Artemis function in cellular response to radiation damage is evident in the hypersensitivity to radiation and persistent cell cycle arrest following radiation damage in cells lacking Artemis [1,16]. To gain insight into the functions of Artemis in cell survival after radiation exposure, we exposed human cells over-expressing Artemis to different qualities of radiation. Our data reveal that elevated Artemis levels provide a distinct survival advantage following exposure to ionizing radiation (IR), and that this effect is more pronounced following high LET exposure as compared to low LET, consistent with an important role for Artemis in resolving complex DNA damage. We find that DNA-PK kinase activity influences the survival advantage imparted by Artemis over-expression to a greater degree that ATM kinase activity and that Artemis phosphorylation is uncoupled from radioresistance.

## Results

### Elevated Artemis protein levels confer high and low LET radioresistance

**Figure 1:**
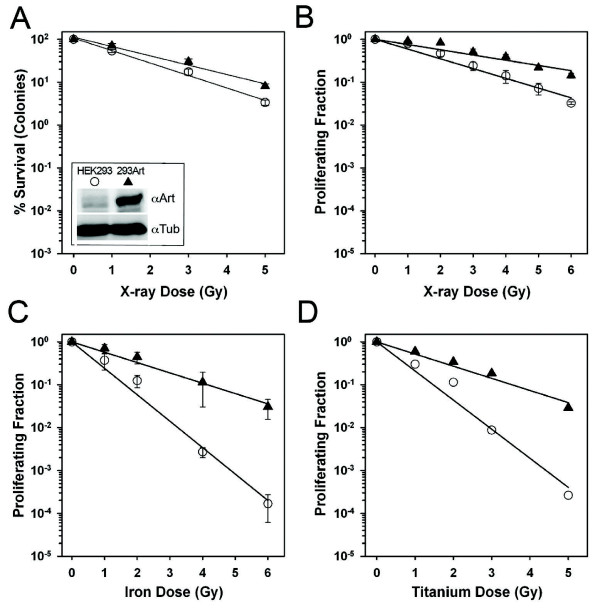
**Elevated Artemis levels confer a radioprotective effect against High and Low LET radiation**. Western blots of lysates from human HEK293 cells carrying integrated vectors harboring tetracycline-inducible Artemis expression constructs (293Art), or control vector (HEK293), probed with antibodies recognizing Artemis (top) or tubulin for loading controls (bottom) (1A-inset). Results from a clonogenic survival experiment using HEK293 (open circles) and 293Art Artemis over expressing cells (solid triangles), stained and colonies scored 14 days after exposure to varied doses of X-rays **(A)** compared to a BrdU proliferation experiment set up in parallel and harvested 48 h post exposure **(B)**. Results from BrdU proliferation experiments following high LET exposures in the form of iron nuclei **(C)** or titanium ions **(D)** carried out using the same dose range as X-ray experiments and processed at the same time points.

To examine the influence of Artemis levels on cellular survival after exposure to high and low LET radiation, we used an extensively characterized human expression embryonic kidney cell line. The wild type line, T-Rex-293 (hereafter, HEK293), expresses native levels of Artemis and the derivative cell line carries a stably integrated doxycycline-inducible Artemis cDNA (hereafter, 293Art) [16] which can be induced to express high levels of Artemis. The T-Rex-293 cell line was chosen for this work due to its ease of transfection, however our studies have shown survival responses after radiation treatments comparable to primary fibroblasts when measured by a standard clonal survival assay and the BrdU proliferation assay (unpublished data). Artemis protein levels were evaluated twenty-four hours after doxycycline treatment by Western blots of whole cell extracts. Artemis protein levels in HEK293 were very low, to non-detectable, whereas 293Art extracts contained high levels of Artemis protein, visualized as an intense band migrating at ~100 kDa on Western blots (Figure 1A inset). Cells stably retain our inducible Artemis expression plasmid and tolerate high levels of induced Artemis expression with no detectable changes in growth, unlike what has been reported for constructs constitutively producing high Artemis levels [18]. Artemis protein levels were similarly validated for all subsequent experiments.

Using these HEK293 and 293Art cell lines we interrogated the role of Artemis in cellular survival after exposure to various radiation qualities. Cellular toxicity has historically been measured with colony formation assays, where cells capable of continued proliferation form colonies that are then stained and counted. To test the effect of Artemis over-expression on radiation sensitivity in human cells we first measured how radiation affected survival in HEK293 versus 293Art cell lines after X-ray exposure using the clonogenic survival assay. Clonal survival data indicate that cells having high levels of Artemis are significantly more resistant to X-rays than the parental cell line having low levels of Artemis (Figure 1A). To further investigate this result, the same cell lines were assayed for X-ray toxicity using a different assay. This assay uses BrdU incorporation into the genome during replication to measure cellular proliferation rather than colony formation. Cells are incubated with BrdU 24 h after radiation and incubated for an additional 24 h prior to harvesting and staining. Thus, cells staining positive for BrdU have initiated DNA synthesis in the 24–48 h after radiation and therefore ‘survived’ radiation treatment. We have used this assay to reproducibly and reliably measure toxicity and cell cycle arrest following both radiation and genotoxic drug treatments in normal and hypersensitive mouse and human lines [11,19,20]. Like the colony formation assay, the BrdU-proliferation assay also reveals increased radioresistance in cells with elevated Artemis protein levels (compare Figure 1A and B).

The BrdU-proliferation assay was then used to evaluate sensitivity to high LET exposures plus and minus Artemis overexpression to assess whether a survival advantage was observed with exposure to a radiation quality that produced greater amounts of complex damage. Two different types of high LET radiation, 1 GeV/u Titanium (LET ~100 keV/u), and 1 GeV/u Fe nuclei (LET ~ 150 keV/u) were used to investigate this point. As observed following X-ray exposure, Artemis over-expression significantly increased the survival of human cells following exposure to high LET radiation (Figure 1C & D). As expected, a markedly higher toxicity was associated with high LET as compared to low LET exposure (Figure 1, compare B, C and D), however Artemis over-expression consistently conferred a significant survival advantage with all radiation qualities. Notably, the degree of radioprotection conferred by Artemis over-expression varied with the radiation quality. The greatest radioprotection was observed after exposure to Fe nuclei (~3 fold), a lesser degree was observed following Ti nuclei (2.2 fold) exposure, and the lowest degree of radioprotection was observed with low LET X-ray exposure (1.7 fold) (Table 1). These data indicate that high levels of intracellular Artemis provide a distinct survival benefit following radiation damage and that the magnitude of this radioprotective effect is radiation quality dependent.

**Table 1:** **Calculation of survival parameters based on proliferation assay using the equation**$\text{Survival}=Exp\left(-\alpha Dose-\beta Dose^{2}\right)$

**A.Survival parameters for 293Art Artemis over-expressing Cells**				
***Radiation Type***	***α, Gy***^**−1**^	***β, Gy***^**−2**^	***D***_**37**_***, Gy***	***Fold***
				***293Art/HEK293c***
X-rays	–	0.057 ± 0.002	4.21 ± 0.087	1.7
Titanium^a^	0.47 ± 0.02	0.034 ± 0.008	1.86 ± 0.024	2.2
Iron^b^	0.22 ± 0.002	0.065 ± 0.007	2.59 ± 0.074	3.0
				
**B.Survival parameters for 293 Wild type Cells**				
**Radiation Type**	***α, Gy***^**−1**^	***β, Gy***^**−2**^	***D***_**37**_***, Gy***	
X-rays	0.287 ± 0.033	0.048 ± 0.006		
Titanium^a^	0.025 ± 0.044	–		
Iron^b^	1.11 ± 0.29	0.086 ± 0.075	0.85 + 0.15	

### High levels of Artemis accelerate DNA double-strand break repair

**Figure 2:**
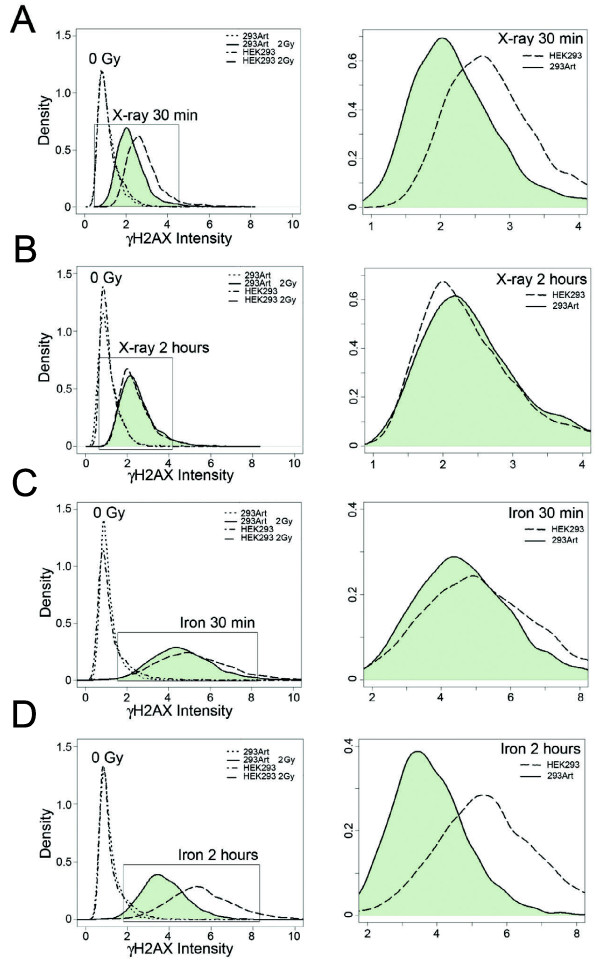
**High levels of Artemis increase the rate of γH2AX resolution in irradiated cells.** Flow cytometry analysis of the distributions of cells with various γH2AX levels in HEK293 and 293Art cells following exposure to different qualities of ionizing radiation. Comparison of the distribution of γH2AX signal in control and irradiated cell populations at various times post exposure (left panels), including an enlarged graph of the irradiated samples (right panels) comparing control HEK293 (dashed lines) and 293Art (shaded). Measurements were taken 30 min after 2 Gy X-rays **(A)**, 2 h after 2 Gy X-rays **(B)**, 30 min after 2 Gy iron nuclei **(C)**, and 2 h after 2 Gy iron nuclei exposure **(D).**

The variant histone H2AX, has been shown to be rapidly phosphorylated at Ser139 (γH2AX) upon induction of DNA DSBs and is a stoichiometric surrogate marker for this type of damage within cells [21,22]. The increase in phosphorylation and disappearance of phosphorylation at this site can be quantified by flow cytometry and faithfully correlates with the kinetics of DSB repair [23,24]. To evaluate the effect of Artemis over-expression on early DNA repair after high and low LET exposures (Fe nuclei and X-ray), we quantified γH2AX phosphorylation in G1 cells by flow cytometry at thirty minutes and two hours after exposure. Individual single-cell flow cytometry data was analyzed and the distribution of γH2AX intensity was quantified and plotted to visualize the DNA damage of cell populations at these time points. As expected, γH2AX intensity increased after ionizing radiation exposure relative to the untreated controls and the levels of damage were greater with Fe nuclei exposure as compared to X-ray (Figure 2, compare A&B to C&D). At thirty minutes after X-ray exposure, cells over-expressing Artemis show significantly less residual damage relative to the parental HEK293 (Figure 2A). Two hours after a 2 Gy X-ray exposure the levels of residual γH2AX are nearly equivalent between the two cell lines (Figure 2B). In contrast, thirty minutes after exposure to 2 Gy of Fe nuclei, HEK293 and 293Art show virtually no difference in γH2AX levels (Figure 2C). However, at two hours post exposure to Fe nuclei the 293Art cells show markedly decreased γH2AX levels as compared to the parental cell line (Figure 2D). These data reveal that Artemis over-expression diminishes γH2AX levels following both high and low LET exposures, but that the kinetics are different between the radiation qualities. These results are similar to previous results in wild type fibroblast cells [23] where high LET radiation results in markedly slower repair kinetics as compared to low LET exposure. Furthermore, these data suggest that the rate of DNA repair is accelerated by Artemis overexpression for both high and low LET radiation damage.

### Artemis phosphorylation is delayed following high LET relative to low LET radiation

**Figure 3:**
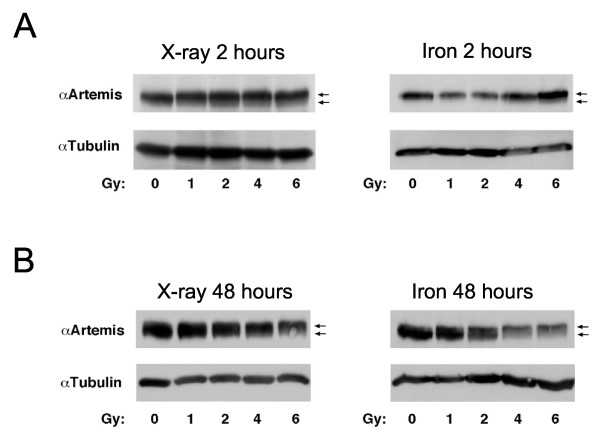
**Artemis phosphorylation is delayed following high LET damage.** Western blots were probed for total Artemis protein and mobility shifts of the protein indicate phosphorylation has occurred. Blots are shown 2 h post X-ray or iron nuclei exposure (**A**, left and right panel respectively) and 48 h post X-ray or iron nuclei exposure (**B**, left and right panel respectively). Arrows indicate location of basally-phosphorylated Artemis (lower arrow) and hyper-phosphorylated Artemis (upper arrow).

Artemis phosphorylation status was evaluated at 2 and 48 h following low and high LET exposures to determine if phosphorylation was temporally regulated with respect to radiation quality. We find that Artemis phosphorylation, which results in a mobility shift of the protein on SDS-PAGE, is delayed following high LET exposure relative to low LET (Figure 3). More specifically, Artemis protein mobility is markedly altered at early time points (2 h) after X-rays while a parallel experiment with high LET radiation shows no change in Artemis protein mobility at this same timepoint (Figure 3A). However, at 48 h after high LET exposure a mobility shift in Artemis protein is observed, indicative of phosphorylation (compare Figure 3A and B). These data point towards a delay in Artemis phosphorylation following high LET as compared to low LET exposure, consistent with our previous studies showing ATM mediated signaling is delayed following high LET relative to low LET exposures in wild type fibroblast cells [23].

### Cells with high Artemis levels show decreased G2/M accumulation following radiation exposure

**Figure 4:**
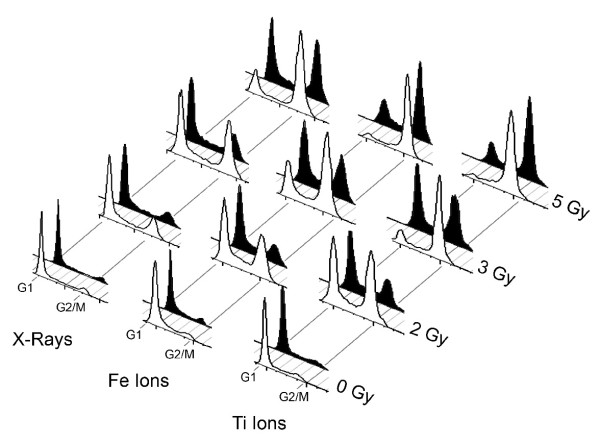
**Artemis over-expression facilitates repair and passage through the G2/M checkpoint.** Flow cytometry of propidium iodine stained cells reveals cell cycle kinetics following various doses of X-ray, Fe ion, or Ti ion exposure. Cell cycle profiles for wild type HEK293 cells are shown in white and cells over-expressing Artemis (293Art) are Black. Peaks corresponding to location of G1 and G2 cells are indicated at the base of the figure, with dose and radiation quality noted on the Y and X axis respectively.

DNA damage initiates cell cycle checkpoints that arrest cell cycle progression until the damage is repaired. To assess the effect of Artemis over-expression on progression through the G2/M checkpoint following radiation, we evaluated cell cycle distributions 48 h after radiation exposure using the DNA stain propidium iodide (PI). Cell cycle distribution was evaluated after exposure to various doses and qualities of ionizing radiation for the 293Art and HEK293 cells (Figure 4).

**Figure 5:**
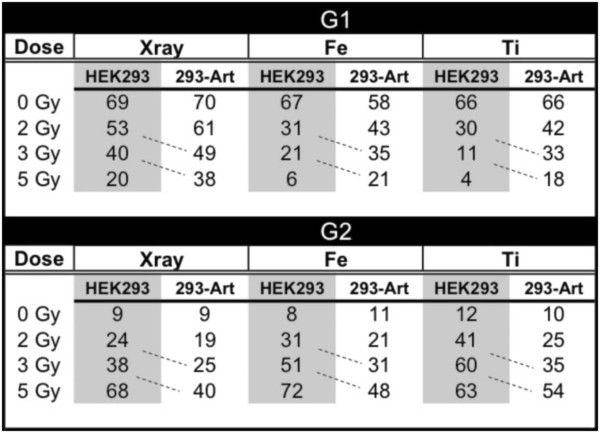
**Quantitation of cell cycle distribution of WT or Artemis over-expressing cells following exposure to varied doses of different radiation qualities.** Normal wild-type (HEK293) or these same cells over-expressing Artemis (293-Art) were exposed to various doses and qualities of radiation. Following radiation cells were incubated for 24 hours, then labeled for 24 hours with BrdU, and fixed at 48 hours post exposure. Flow cytometry was used to quantify the percentage of cells in G1 or G2.

High Artemis levels had no discernable effect on cell cycle distributions in the absence of DNA damage. As expected, both 293Art and HEK293 cells showed a marked accumulation of cells at the G2/M boundary following radiation exposure and this accumulation increased at higher doses, and was greater with high LET as compared to low LET exposure (Figure 4). In previous studies with Artemis defective cells we observed persistent cell cycle arrest and an absence of cycling 48 h post radiation exposure [16], in contrast, cells over-expressing Artemis traverse the G2/M checkpoint more readily and move into G1 to a greater extent than control wild type HEK293 (Figure 4). Similar results are also observed at 72 h post radiation exposure suggesting that the proliferation defect in wild type cells is due to a damage-induced cell cycle block, which is not rectified even at 72 h after irradiation (not shown). Importantly, Artemis over-expression does not eliminate or bypass the G2/M cell cycle checkpoint, but rather cells with elevated Artemis levels have an attenuated biological response to irradiation with cell cycle kinetics similar to the parental line albeit at a lower dose (i.e. cell cycle profile of HEK293 at 2 Gy is comparable to 293Art at 3 Gy, Figure 4). This effect was observed at different doses and with different radiation qualities (Fe and Ti), rendering the cell cycle distribution of 293Art cells at higher doses nearly identical to that of the parental HEK293 at lower doses (Figure 5, note patterns highlighted by dotted lines).

Artemis over-expression consistently resulted in a larger percentage of cells cycling into G1 with fewer cells remaining arrested at G2/M after exposure, a phenomenon most consistent with an increased DNA repair capacity or repair rate.

### ATM and DNA-PK regulate the radioresistance conferred by Artemis

**Figure 6:**
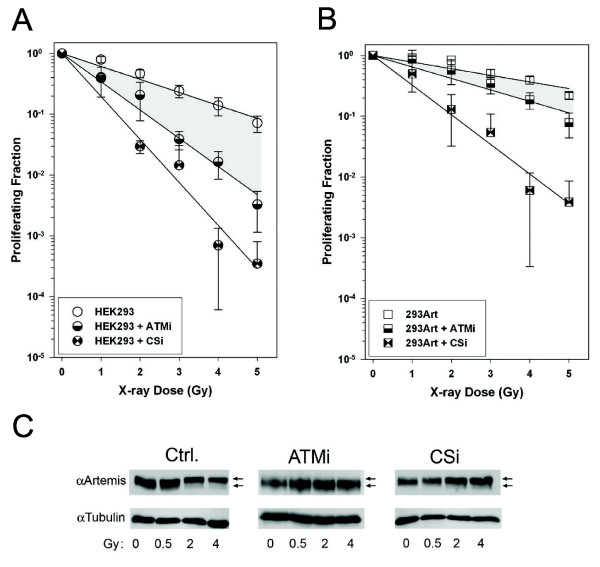
**Effect of kinase inhibitors on the survival advantage imparted by Artemis over-expression and Artemis phosphorylation following X-ray exposure.** BrdU proliferation assays were performed on both wild type HEK293 **(A)** and cells over-expressing Artemis **(B)** with and without two different kinase inhibitors, a ATM kinase inhibitor (ATMi) and a DNA PKcs inhibitor (CSi). Shaded regions on these graphs are for visual comparison of the effect ATM inhibition on the ability of wild-type versus Artemis over-expressing cells to proliferate post X-ray exposure. Artemis phosphorylation is indicated by a shift of the total Artemis protein on a Western gel (arrows indicate basally and hyper-phosphorylated forms) following various doses of X-ray and was also monitored plus ATM inhibition **(C)** and DNA PKcs inhibition **(D).**

Both DNA-PK and ATM are reported to modify Artemis and to be important mammalian DNA damage signaling molecules. We next evaluated the functional significance of these kinase activities on the radioprotective effect imparted by Artemis over-expression. We carried out cell proliferation assays to quantify the survival advantage of Artemis over-expression under conditions where ATM or DNA-PK were catalytically inactivated with inhibitory drugs at a 10 μM concentration, previously shown to totally block radiation induced phosphorylation of down stream targets [25,26]. As expected, the parental HEK293 cells were significantly more sensitive to X-ray treatments when either ATM or DNA-PK was inactivated with specific kinase inhibitors (Ku55933, or Nu7026 respectively). Under these experimental conditions, sensitivity to radiation was greater when DNA-PK was inhibited relative to inhibition of ATM (Figure 6A). The survival of 293Art cells was likewise influenced by the inhibition of ATM and DNA-PK (Figure 6B), however the effect of ATM kinase inhibition was significantly less pronounced when Artemis levels were elevated (compare 6A & B, shaded areas). In contrast, DNA-PK kinase inhibition reduced survival dramatically in Artemis over-expressing cells, approximately two orders of magnitude at the highest dose (Figure 6B), similar to what was observed in wild type cells with DNA-PK inhibition (compare 6A & B). These data imply that the radioprotective effect conferred by elevated Artemis is significantly more dependent on DNA-PK than ATM activity. In addition, these data suggest that the mechanism of the radioprotection resulting from Artemis over-expression involves a coordinate activity of Artemis and DNA-PK, most likely in enhancing DNA repair.

The kinase dependency of Artemis phosphorylation following radiation exposure was also examined. Multiple kinases could potentially phosphorylate Artemis, therefore in order to assess DNA-PK and ATM activity on Artemis on phosphorylation levels Western blots of 293Art cells two hours post X-ray exposure (0 Gy, 0.5 Gy, 2 Gy and 4 Gy) were run to assess the phosphorylation status of Artemis in the presence and absence of inhibitors. In the absence of ATM inhibitor, Artemis shows a dose dependent increase in phosphorylation, visualized as a shift in mobility of the band specific for Artemis protein (Figure 6C, control). Inhibiting ATM kinase blocks the induction of Artemis phosphorylation following radiation as exhibited by a lack of shifted Artemis protein on Western blots (Figure 6C, ATMi). Treatment of cells with the DNA-PKcs inhibitor (CSi) only partially blocked the mobility shift of Artemis, with cells showing less phosphorylation and lowered levels of shifted protein (Figure 6C). The latter result suggests that this phosphorylation is uncoupled from the survival advantage imparted by Artemis over-expression, as inhibition of DNA PK kinase severely affected the radioprotective effect but did not affect Artemis phosphorylation. Furthermore, these data indicate that the mobility shift associated with Artemis phosphorylation is primarily ATM dependent whereas the radioprotective effects of Artemis are predominantly DNA-PK dependent.

## Discussion

Previous studies by our group and others have shown that Artemis deficient cells exhibit elevated radiation sensitivity, yet have only a slight defect in DSB repair, implicating a role for Artemis in the repair of a specific class of DSBs [6,13,16]. Radiation-induced breaks consist of a combination of closely spaced single-strand breaks, DSBs, sugar damage, base damage and breaks with modified ends. Only specific subsets of these breaks are thought to be processed by Artemis, with other proteins apparently unable to substitute for Artemis in this function [10,11,13]. In G1 cells, the majority of radiation-induced DSBs are rejoined quickly by NHEJ and this rapid repair appears not to require Artemis or ATM, however a small subset of slow repairing damages have been shown to require ATM and Artemis for repair [13,27,28]. Interestingly, a recent report outlines a requirement for ATM and Artemis in the repair of a fraction of breaks refractory to repair by homologous recombination in G2 cells as well [29].

Neither the precise role for Artemis in DSB repair nor its substrate specificity *in vivo* has been completely defined. Some studies have suggested a specific role for Artemis in the repair of complex DSBs, however it’s exact role in repairing damage caused by high LET radiation remains unclear [13,30]. The current study focuses on identifying the contribution of Artemis to the repair of various radiation qualities. In these studies we show that Artemis over-expression provides a survival advantage post Iron exposure. Data from a representative experiment with Titanium exposure shows a similar pattern to Iron, again confirming that Artemis protein is more protective following high LET exposures and that the level of protection is dependent on radiation quality. Western blot analysis (Figure 1A insert) verifies the dramatically increased Artemis levels in 293Art cells in comparison to wild type HEK293 cells and further confirms the correlation between the increased radioresistance and increased Artemis expression observed in these cells. High Z particles like Fe and Ti are not commonly used in cancer therapeutic regimens as the LET of these particles in normal tissue at the entrance and prior to the target site is very high. Typically, low charge particles like carbon ions are used in particle therapy in centers offering high LET therapies (such as the Heidelberg Ion Therapy Centre (UKL-HD); the Heavy Ion Medical Accelerator in Chiba, Japan; the CNAO facility near Milan in Pavia; and GSI in Darmstadt, Germany). The suitability of carbon is mainly attributed to its low LET (13 keV/micron) at the entrance of the tissue and substantial Bragg peak (several hundred keV/micron) at the site of the tumor. We have used Ti and Fe ions in these studies as these ion/energy combinations have a LET similar to that of the carbon ion beam near the Bragg peak and therefore mimic the high LET deposited at the tumor site during therapy.

Studies of γH2AX levels after damage reveal more rapid resolution of phosphorylated H2AX in cells over-expressing Artemis. While elevated Artemis levels reduced the γH2AX signal in cell populations after both high and low LET damages, the timing of these differences is distinctly different. Artemis-mediated reduction in γH2AX levels (indirectly corresponding to reduced levels of DSBs) following high LET radiation exposures is only marginally detectable thirty minutes after exposure, however a clear effect is observed at two hours post exposure (compare Figure 2C & D). In contrast, low LET damage shows a dramatic difference in γH2AX levels between Artemis over-expressing and wild type HEK293 cells at thirty minutes, whereas at two hours post exposure the control and Artemis over-expressing cells are nearly indistinguishable (compare Figure 2A & B). This likely reflects a slower repair of the more complex damage induced by high LET radiation as compared to less complex low LET radiation damage as has been previously observed [23,31]. Nonetheless, these data show that Artemis levels significantly influence γH2AX resolution and likely the repair kinetics following both high and low LET radiation.

Inhibition of DNA-PK reveals that the radio-protective effect of Artemis over-expression was principally reliant on DNA-PK activity and to a lesser degree on ATM activity. In contrast, we find that Artemis phosphorylation, indicated by a mobility shift of Artemis protein, was primarily dependent on the activity of ATM, as has been reported previously [14,17,32]. Furthermore, in cells containing high levels of Artemis, cell proliferation following irradiation was more severely diminished by loss of DNA-PK activity than by ATM inhibition (compare Figure 6A & B). Taken together, these data suggest that Artemis phosphorylation as observed by mobility shifts may not be relevant to Artemis’ function in the repair of radiation damage, as we had previously suggested [16].

## Conclusions

In summary, our data reveal that elevated Artemis levels in human cells are able to confer a statistically significant survival advantage following exposure to ionizing radiation. Moreover, this Artemis-mediated radioprotective effect is more pronounced following high LET exposures as compared to low LET damage (Figure 1 and Table 1). We also find that Artemis over-expression accelerates the resolution of γH2AX, and by inference the rate of double-strand break repair *in vivo* (Figure 2). This latter finding is entirely consistent with our observation that high levels of Artemis increase the rate at which cells are able to satisfy the G2/M checkpoint after radiation damage (Figure 4). In addition, we find that Artemis-radioprotection is primarily dependent on DNA-PK activity and not coupled to the ATM-dependent phosphorylation (Figure 5). Here we find that elevated Artemis levels alone can significantly alter DNA repair and cellular survival following radiation exposures. Our data are most consistent with Artemis functioning in the repair of complex DNA breaks as part of the NHEJ repair pathway and suggest that that Artemis is probably rate limiting to this process within human cells. We have observed increased Artemis levels in a subset of breast tumors, although we did not find this to be the singular determinant of radiosensitivity (unpublished data). These findings suggest that Artemis levels in a subset of tumor cells may be a useful indicator of responsiveness to radiation therapy. Moreover, Artemis inhibition may provide a functional adjuvant in radiation therapy in such cases, a possibility we are currently investigating.

## Methods

T-Rex-293 (Invitrogen) human kidney cells employed in these studies have a rapid doubling time, high efficiency of transfection and protein expression, accurate translation and post translational folding and processing to generate functional proteins and have been extensively used as an expression tool for recombinant proteins in the last three decades. These cells expressing the tetracycline repressor were cultured in DMEM supplemented with 10% tetracycline-free FBS and 5 mg/ml of blasticidin. T-Rex-293 cells were transfected with pcDNA4/TO/Myc-HisA (Invitrogen) carrying wild type Artemis cDNA, incubated for 48 h and then placed under zeocin selection (250 μg/ml). Clones were isolated, induced for Artemis expression with 1 μg/ml doxycycline 24 h prior to radiation exposure, this induction is maintained during the course of the experiment and Artemis expression levels were evaluated by immunoblotting. For both γH2AX flow cytometry and the BrdU proliferation assay, T-25 flasks were plated for each dose and cells were grown to 85 to 95% confluence prior to exposure.

### Irradiation conditions

Cells were exposed to doses ranging from 1 to 6 Gy of 320 kV/X-rays, 1,000 MeV/nucleon Fe, or 1,000 MeV/nucleon Ti ions (LET of 150 keV/μm and 108.5 keV/μm respectively). X-ray exposures were performed at Lawrence Berkeley National Laboratory using a Pantak® X-ray generator operating at 320 kV/10 mA with a 0.5 mm copper filtration and using dose rates ranging from 5 to 80 cGy/min depending on the dose. Fe and Ti ion exposures were performed at Brookhaven National Laboratory, NY using dose rates ranging from 10 cGy to 1 Gy/min depending on the dose. Standard radiation therapy regimens typically follow a fractionated regimen of 1.5–2 Gy doses to a cumulative dose of 40–60 Gy, depending on nature of the tumor. Radiation doses used in this study are comparable to single fraction, or simulate an accumulated dose from 2–3 fractions.

### Kinase inhibitor treatments

Cells were exposed to an ATM kinase inhibitor (Calbiochem, EMD Biosciences, Gibbstown, NJ) and/or a DNA-Dependent Protein Kinase inhibitor (NU7026 SigmaAldrich, St. Louis, MO) in a subset of experiments to define the relative role of these kinases in imparting the survival advantage in cells over-expressing Artemis (293Art). For these experiments cells were pre-incubated with these inhibitors using a 1 μM concentration for 1 h prior to irradiation.

### Clonogenic survival assay

For colony formation assays, varying number of cells (1.5 × 10^2^ to 1 × 10^4^) were plated in triplicate 60 cm^2^ plastic dishes and incubated for 12–15 h at 37°C, 5% CO_2_ prior to irradiation. Cells were irradiated with X-ray in the dishes and returned to the incubator for 7–8 days. Resulting colonies were stained with crystal violet, scored, and surviving fraction calculated and plotted.

### BrdU proliferation assay

Cells were irradiated with high or low LET radiation, incubated for 24 h then labeled with BrdU for 24 h, and finally fixed and stained 48 h after exposure using an anti-BrdU antibody (20 μl antibody per 1 × 10^6^ cells, BD Biosciences, San Jose, CA) following product directions. Cell cycle distribution and BrdU incorporation were determined by data collected with a Beckman-Coulter EPICS XLMCL flow cytometer using Cellquest Data Acquisition software, and analyzed using Flowjo (Treestar, Inc. OR) software. Percentage of G1 cells incorporating BrdU were determined for each dose and cell line, and values graphed relative to controls.

### Calculation of survival parameters following radiation exposure

D_0_ gives the mean lethal dose that delivers an average of one lethal event/target. When D = D_0_ the surviving fraction is 37%. We calculated the D_37_ values for each cell line and radiation quality, and found them to be consistently lower in wild type HEK293 cells as compared to the Artemis over-expressing cell line, 293Art. Alpha and beta components were also evaluated for each cell line following each radiation quality on the basis of the equation: $\text{S}={\text{e}}^{\wedge }\left(-\alpha \text{D}-\beta \text{D}2\right)$, where S is the survival fraction, D is the dose of exposure and α and β are two numerical constants. The alpha term is said to dominate at low dose [linear with dose for log (survival)] and to be due to single ions or electrons. The beta term is more prevalent at high dose [quadratic with dose for log (survival)] and said to arise from two or more tracks or miss-repair that increases with dose. If alpha is zero, or very small, then cells are resistant and only beta contributes.

### Flow cytometry analysis of γH2AX kinetics

Irradiated and mock-irradiated flasks were fixed at various times after exposure. Cells were harvested and fixed with 100% ice-cold methanol added drop-wise while vortexing. After fixation, cells were placed at −20°C until stained. Cell suspensions were counted prior to staining and diluted to 1.0 × 10^6^/ml. For staining, an aliquot of the fixed cells containing 0.5 × 10^6^ was removed and washed in PBS. Cells were placed in a blocking solution containing 2% FBS/PBS and incubated for 1 h with a primary mouse monoclonal γH2AX antibody (1:800 dilution) from Molecular Probes (Invitrogen, Carlsbad, CA) on ice. After incubation, cells were pelleted, washed in PBS, and incubated in a secondary goat anti-mouse antibody (1:400 dilution) from Molecular Probes (Invitrogen, Carlsbad, CA) diluted in blocking solution. After 1 h additional incubation on ice under foil, cells were pelleted and washed for a final time in PBS. Flow cytometry was used to define cell cycle status by staining cellular DNA with propidium iodide (10 μg/ml PI, Roche Applied Science, Indianapolis, IN) in a solution containing RNase (100 μg/ml, Sigma-Aldrich, St. Louis, MO). Median values of fluorescent signals corresponding to γH2AX levels were obtained for each control and irradiated sample (X-ray or 1,000 MeV/u Fe ion). Cell data was collected on a Beckman-Coulter EPICS XLMCL flow cytometer using Cell quest Data Acquisition software, and analyzed using Flowjo (Treestar, Inc. OR) software. Individual cell data was exported for analysis and data is expressed as relative increase in phosphorylation level over controls for each experiment.

### Western blots

Cells were harvested and resuspended in cold high salt lysis buffer (20 mM Tris, pH 8.0; 600 mM NaCl; 1 mM EDTA; 0.5% Igepal) containing protease inhibitor cocktail from Calbiochem (1 mM PMSF, 1 mM Na3VO4, and 1 mM NaF), incubated on ice for 30 min with occasional vortexing, and then sonicated. Lysates were spun at 14,000 rpm for 20 min at 4°C, and protein concentration determined using the Biorad BCA protein assay. Loading buffer (187.5 mM Tris, pH 6.8; 6% SDS; 30% Glycerol, and 10% 1 M DTT) was added to samples, which were boiled and spun down. Samples were loaded onto a 7% SDS-PAGE gel and run for 1 h at 150 V (Running buffer: 25 mM Tris, 192 mM Glycine, 0.1% SDS). Proteins were transferred to 0.2um PVDF membrane at 4°C, 30 V, over night in Transfer Buffer (20% MeOH, 50 mM Tris, 400 mM Glycine, 0.1% SDS). After the transfer, blots were rinsed with Tris buffered saline containing Tween 20 (TBST), then blocked with 5% BSA-TBST for 1 h prior to antibody addition. Primary antibodies were incubated at each dilution and time specified: Tubulin (1:5,000 for 1 h, Sigma-Aldrich, St. Louis, MO) and total Artemis 1024 anti-sera (1:1,000 for 1 h). A description of total Artemis anti-sera production was described previously [16]. Briefly, a ~600 bp fragment of Artemis was sub-cloned into pET-15b (Novagen) vector and expressed in *E. coli*. Artemis protein from these cells was then purified according to the manufacturers directions (pET System Manual, Novagen). Purified protein was used to generate rabbit antiserum (Bethyl Laboratories). Secondary antibodies were either anti-mouse-HRP from GE Healthcare (1:15,000 or 1:10,000; 1 h) or anti-rabbit-HRP from Cell Signaling (1:5,000 or 1:10,000; 1 h). Blots were washed for 3 × 10 min after the primary and secondary incubations with TBST. ECL Substrate was either ECL Supersignal West Pico from Pierce or ECL Plus from GE Healthcare.

## Abbreviations

DNA-PK: DNA protein kinase; ATM: ataxia telangiectasia mutated; NHEJ: Non-homologous end joining; ATR: ataxia telangiectasia- and Rad3-related.

## Competing interests

The authors declare that they have no competing interests.

## Authors’ contributions

DMS aided in culturing cells, performed irradiations, helped with fixations for proliferation assay and drafted the manuscript. JY carried out the immunoassays. MKW aided with culturing of cells, irradiations, fixation for proliferation assay, flow cytometry staining and analysis. DA perfomed Western blot analysis and helped to draft the manuscript. FAC performed the statistical analysis and calculation of survival parameters based on the proliferation assay results and participated in the editing of the manuscript. MK performed the clonal survival experiment, participated in editing, and provided intellectual feedback on the manuscript. SMY helped to conceive the study, helped in the drafting and editing of the manuscript, and produced figures based on the data provided for the paper. JMP helped to conceive the study, designed the study, performed portions of all of the technical assays, coordinated efforts, participated in the drafting and editing of the manuscript, and finalized the final version. All authors read and approved the final manuscript.
